# A New Triterpenoid from *Teucrium viscidum*

**DOI:** 10.3390/molecules18011262

**Published:** 2013-01-21

**Authors:** Xincai Hao, Jinwen Zhang, Guangxin Xia, Yongbo Xue, Zengwei Luo, Yanyan Si, Guangmin Yao, Yonghui Zhang

**Affiliations:** 1 Hubei Key Laboratory of Natural Medicinal Chemistry and Resource Evaluation, School of Pharmacy, Tongji Medical College, Huazhong University of Science and Technology, Wuhan 430030, China; 2 Tongji Hospital Affiliated to Tongji Medical College, Huazhong University of Science and Technology, Wuhan 430030, China; 3 Central Research Institute, Shanghai Pharmaceutical Holding Co. Ltd, 898 Halei Road, Zhangjiang Hi-Tech Park, Shanghai 201203, China

**Keywords:** *Teucrium viscidum*, triterpenoids, lignans, 11*β*-HSD1

## Abstract

A new ursane-type triterpenoid, 3*β*-hydroxy-urs-30-*p*-*Z*-hydroxycinnamoyloxy-12-en-28-oic-acid (**1**), together with three known triterpenoids, 3*β*-hydroxy-urs-30-*p*-*E*-hydroxycinnamoyloxy-12-en-28-oic-acid (**2**), 2*α*,3*β*,19*α*-trihydroxy-urs-12-en-28-oic-acid (**3**), and ursolic acid (**4**), four known lignans, pinoresinol (**5**), 9*α*-hydroxypinoresinol (**6**), (+)-medioresinol (**7**), and (+)-kobusin (**8**), and two steroids, *β*-sitosterol (**9**), and daucosterol (**10**), were isolated from the whole parts of *Teucrium viscidum*. Their structures were established by a combination of spectroscopic data analysis, besides comparison with literature data. Compounds **1**–**4** were evaluated for their inhibitory activities against 11*β*-hydroxysteroid dehydrogenase 1 (11*β*-HSD1).

## 1. Introduction

**Figure 1:**
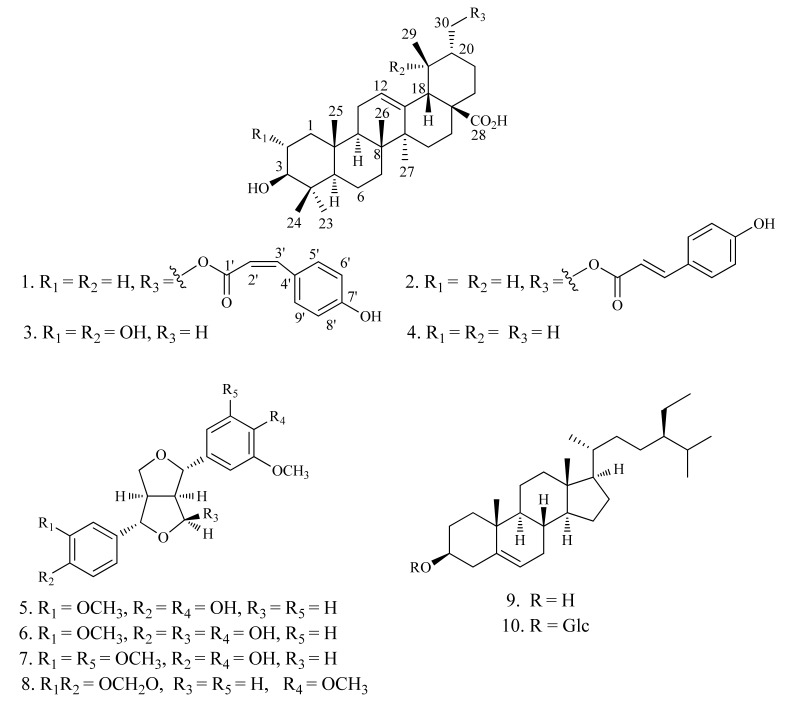
Structures of compounds **1**–**10**.

*Teucrium viscidum* Blume (Lamiaceae), known as Shan-Huo-Xiang in China, is an annual herb, whose whole parts are used as a traditional Chinese medicine to treat hemoptysis, hematemesis, pulmonary abscesses, traumatic injuries, and bites of rabies-stricken dogs or venomous snakes [1]. Previous phytochemical studies on *T. viscidum* have resulted in the isolation of several neoclerodane diterpenoids [2,3,4,5]. In the course of search for novel natural products from traditional Chinese medicines, a new triterpenoid **1**, together with nine known compounds **2**–**10**, were isolated from the whole parts of *T. viscidum*. In this paper, we report the isolation and structural elucidation of compounds **1**–**10** (Figure 1), as well as the inhibitory activities against 11*β*-HSD1 of compounds **1**–**4**.

## 2. Results and Discussion

The acetone extract of the air-dried whole parts of *T. viscidum* was suspended in water and successively partitioned with petroleum ether, CHCl_3_, and EtOAc. The CHCl_3_ fraction was subjected to column chromatography to afford one new triterpenoid **1**, along with nine known compounds, 3*β*-hydroxy-urs-30-*p*-*E*-hydroxycinnamoyloxy-12-en-28-oic-acid (**2**) [6], 2*α*,3*β*,19*α*-trihydroxy-urs-12-en-28-oic-acid (**3**) [7], ursolic acid (**4**) [7], pinoresinol (**5**) [8], 9*α*-hydroxypinoresinol (**6**) [9], (+)-medioresinol (**7**) [10], (+)-kobusin (**8**) [11], *β*-sitosterol (**9**) [12], and daucosterol (**10**) [12]. The known compounds were identified on the basis of NMR spectroscopic analyses and comparison with those reported in the literature (Figure 1).

Compound **1** was obtained as an amorphous powder. Its molecular formula, C_39_H_54_O_6_, could be deduced from the (+)-HRESIMS peak at *m/z* 641.3799 [M+Na]^+^ (calcd. for C_39_H_54_O_6_Na^+^, 641.3818). The IR spectrum showed absorption bands for hydroxyl (3729 cm^–1^), carbonyl (1685 cm^–1^) and benzene ring (1602 cm^–1^) functions. The ^1^H-NMR spectrum of **1** (Table 1) showed five tertiary methyl singlets at *δ*_H_ 0.92 (3H, s), 1.05 (3H, s), 1.08 (3H, s), 1.23 (3H, s), and 1.26 (3H, s), one secondary methyl doublet protons at *δ*_H_ 1.09 (3H, d, *J* = 6.0 Hz), three protons attached to oxygenated carbons at *δ*_H_ 4.50 (1H, dd, *J* = 3.2 Hz, 11.2 Hz), 4.27 (1H, dd, *J* = 7.2 Hz, 11.2 Hz), 3.48 (1H, dd, *J* = 6.8 Hz, 9.2 Hz), three olefinic protons at *δ*_H_ 7.03 (1H, d, *J* = 12.8 Hz), 6.09 (1H, d, *J* = 12.8 Hz), 5.52 (1H, t, *J* = 3.2 Hz), and a *p*-disubstituted benzene ring at *δ*_H_ 8.09 (d, *J* = 8.4 Hz, 2H) and 7.22 (d, *J* = 8.4 Hz, 2H). The ^13^C-NMR, DEPT, and HSQC spectra for **1** exhibited thirty nine carbon signals differentiated as six methyls, ten methylenes (including an oxygenated), thirteen methines (including seven olefins and an oxygenated), and ten quaternary carbons (including two carbonyls, an olefin, and an oxygenated).

**Figure 2:**
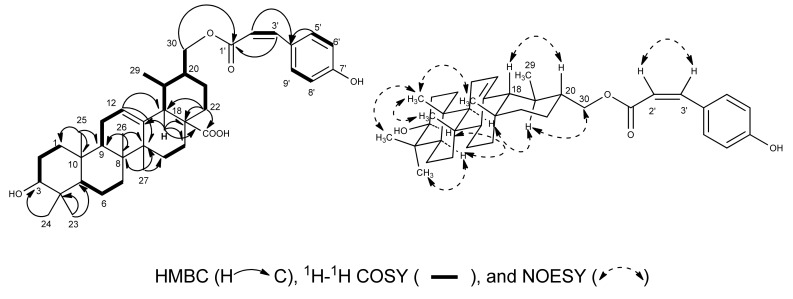
^1^H-^1^H COSY, Key HMBC, and NOESY correlations of compound **1**.

The NMR data of **1** were quite similar to those of 3*β*-hydroxy-urs-12-en-28-oic-acid (**3**), except for signals for a nine-carbon chain [7,13] which was composed of one carbonyl (*δ*_C_ 167.5), one double bond (*δ*_H_ 6.09, d, *J* = 12.8 Hz, H-2'; *δ*_H_ 7.03, d, *J* = 12.8 Hz, H-3'; *δ*_C_ 116.4, C-2'; *δ*_C_ 144.5, C-3') and *p*-substituted phenol (*δ*_H_ 8.09, d, *J* = 8.4 Hz, H-5', H-9'; *δ*_H_ 7.22, d, *J* = 8.4 Hz, H-6', H-8'; *δ*_C_ 134.0, C-5', C-9'; *δ*_C_ 116.5, C-6', C-8', *δ*_C_ 161.7, C-7'). In the HMBC spectrum, the cross peaks of these two newly olefinic protons H-2' and H-3' to the conjugated carbonyl C-1' (*δ*_C_ 167.5) and the aromatic carbon C-4' (*δ*_C_ 127.1) of the *p*-substituted phenol, as well as the aromatic protons H-5'/H-9' of the *p*-substituted phenol to the newly olefinic carbon C-3', revealed that this double bond was fixed between the carbonyl and the *p*-substituted phenol, suggested the presence of a *p*-hydroxycinnamoyl group in **1**. The coupling constant *J*_H-2',H-3'_ = 12.4 Hz of H-2' and H-3', and the NOESY correlation between H-2' and H-3', indicated the *Z*-geometry of the double bond in the *p*-hydroxycinnamoyl group. The HMBC correlation of H-30 (*δ*_H_ 4.50, dd, *J* = 3.2 Hz, 11.2 Hz, H-30a; *δ*_H_ 4.27, dd, *J* = 7.2 Hz, 11.2 Hz, H-30b) to the carbonyl C-1' of the *p*-*Z*-hydroxycinnamoyl group suggested the *p*-*Z*-hydroxycinnamoyl group was connected to 30-OH. HSQC, ^1^H–^1^H COSY, HMBC, and NOESY analysis (Figure 2) allowed us to assign compound **1** as 3*β*-hydroxy-urs-30-*p*-*Z*-hydroxycinnamoyloxy-12-en-28-oic-acid.

The 1 type of 11β-hydroxysteroid dehydrogenase (11β-HSD 1) has been recognized to be an attractive target in metabolic disease or type 2 diabetes, and some distinct 11β-HSD 1 inhibitors have entered clinic trials [14]. Since pentacyclic triterpenoids were reported to be potential inhibitors of 11*β*-HSD 1 [15], compounds **1**–**4** were tested for their inhibitory activities against human 11*β*-HSD 1 *in vitro*. Ursolic acid (**4**) showed stronger inhibitory activity on 11*β*-HSD1, with IC_50_ values of 1.5 μM, which was in accordance with the reported value [15]. Compound **3** displayed exceedingly weak inhibitory activity on 11*β*-HSD1 by comparison of the other triterpenoids in our assay. Compounds **1** and **2** did not show obvious activity against 11*β*-HSD 1, which suggested that the variation of CH_3_-30 could reduce 11*β*-HSD 1 inhibitory activity. Structure-activity relationships of pentacyclic triterpenoid inhibitors of 11*β*-HSD 1 showed that the hydroxyl group at position C-19 could decrease 11*β*-HSD 1 inhibitory activity [15]. Our bioassay not only confirmed this conclusion, but also indicated that the variation of CH_3_-30 could also reduce the 11*β*-HSD 1 inhibitory activity. These results significantly extend our knowledge of the structure-activity relationship of ursolic acid derivatives as 11*β*-HSD 1 inhibitors.

## 3. Experimental

### 3.1. General Procedures

Optical rotations were measured on a Perkin Elmer PE-341LC polarimeter. IR spectra were recorded as KBr disks on a Bruker Vertex 70 FT-IR spectrophotometer. HRESI-MS data were measured on an API QSTAR Pulsar spectrometer. NMR spectra were recorded using a Bruker AM-400 spectrometer, and the ^1^H-NMR and ^13^C-NMR chemical shifts were referenced to the solvent peaks for C_5_D_5_N at *δ*_H_ 8.74, 7.58, 7.22, and *δ*_C_ 150.35, 135.91, 123.87. Silica gel (200–300 mesh, Qingdao Marine Chemical Inc., Qingdao, China), Amberchrom CG161M (75 μm, Rohm and Haas Company, Philadelphia, PA, USA), ODS (50 μm, YMC, Kyoto, Japan), and Sephadex LH-20 (Pharmacia Biotech AB, Stockholm, Sweden) were used for column chromatography. HPLC separation was performed on an instrument consisting of an Agilent 1100 controller, an Agilent 1100 pump, and an Agilent UV detector with an YMC (250 × 10 mm, 5 μm) preparative column. TLC was carried out on precoated silica gel GF_254_ plates. Spots were visualized under UV light (254 or 356 nm) or by spraying with 5% H_2_SO_4_ in 95% EtOH followed by heating.

### 3.2. Plant Material

The whole parts of *T. viscidum* were collected at Shiyan, Hubei Province, China, in June 2010, and identified by Prof. Changgong Zhang of School of Pharmacy, Tongji Medical College, Huazhong University of Science and Technology. The voucher specimen (No. 2010-0616) was deposited in the herbarium of Hubei Key Laboratory of Natural Medicinal Chemistry and Resource Evaluation, School of Pharmacy, Tongji Medical College, Huazhong University of Science and Technology.

### 3.3. Extraction and Isolation

The dried whole parts of *T. viscidum* (50 Kg) were extracted with 70% acetone (100 L × 3 times) at the room temperature. After removal of the solvent, the crude extraction (2.3 kg) was suspended in H_2_O (5.0 L) and partitioned successively with petroleum ether (60–90 °C), CHCl_3_, and EtOAc to give petroleum ether, CHCl_3_, and EtOAc portions. The CHCl_3_ portion (200 g) was subjected to MCI gel column eluted with MeOH/H_2_O (9:1, v/v), and then chromatographed on a silica gel column eluted with petroleum–acetone (50:1 to 0:1, v/v) to obtain seven fractions (A–G). Compound **9** (40 mg) was recrystallized from fraction A using EtOAc. Compound **4** (100 mg) was obtained by recrystallization in EtOH from fraction B. Fraction C was subjected to a Sephadex LH-20 column eluted with MeOH to give four subfractions (C_1_–C_4_). Subfraction C_2_ was purified by semi-preparative HPLC (70% MeCN in H_2_O, flow rate 1.5 mL/min, wavelength 254 nm) to obtain compounds **2** (5.0 mg, retention time 30 min) and **1** (6 mg, retention time 33 min). Subfraction C_3_ was subjected to ODS column chromatography eluted with 80% MeOH in H_2_O, and then purified by semi-preparative HPLC (85% MeOH in H_2_O, flow rate 1.5 mL/min, wavelength 254 nm) to yield compound **3** (7 mg, retention time 45 min). Fraction D was applied to RP-C_18_ gel column eluted with MeOH/H_2_O (1:5 to 1:0, v/v), followed by chromatography over repeated silica gel column (CHCl_3_/MeOH, 60:1, v/v) to afford compound **5** (8 mg). Fraction E was subjected to RP-C_18_ gel column eluted with MeOH/H_2_O (1:5 to 1:0, v/v), followed by chromatography over silica gel and finally purified by semi-preparative HPLC (40% MeOH in H_2_O, flow rate 1.5 mL/min, wavelength 205 nm) to get compounds **6** (6 mg, retention time 43 min ) and **7** (9 mg, retention time 46 min). Fraction F was subjected to a Sephadex LH-20 column eluted with MeOH, followed by chromatography over silica gel eluted with petroleum/acetone (15:1, v/v) to get compound **8** (10 mg). Compound **10** (100 mg) was directly crystallized from fraction F.

*3β-hydroxy-urs-30-p-Z-hydroxycinnamoyloxy-12-en-28-oic-acid* (**1**). Amorphous powder. [α${]}_{D}^{25}$: + 27 (*c* = 0.85, THF); UV (THF) *λ*_max_ (log*ε*) nm: 312 (3.33), 208 (3.39); IR (KBr) ν_max_ 3729, 2924. 1685, 1601, 1512, 1457, 1384, 1260, 1024, and 772 cm^–^^1^; ^1^H-NMR (C_5_D_5_N, 400 MHz) and ^13^C-NMR (C_5_D_5_N, 100 MHz) see Table 1; HRESIMS *m/z*: 641.3799 [M+Na]^+^ (calcd. for C_39_H_54_O_6_Na^+^, 641.3818).

### 3.4. 11β-HSD1 Inhibitory Assays

The scintillation proximity assay (SPA) was used to analyze the inhibitory activities of compounds **1**–**4** on human 11*β*-HSD1. Microsomes containing 11*β*-HSD1 were used according to the reported studies [16]. The full-length cDNAs of human 11*β*-HSD1 were isolated from the cDNA libraries provided by NIH Mammalian Gene Collection and cloned in pcDNA3 expression plasmid. HEK-293 cells were transformed with the pcDNA3-derived expression plasmid. Transformed cells were screened by 700 μg/mL G418. The microsomal fraction, which stably expressed 11*β*-HSD1, was prepared from the HEK-293 cells, and then was used for SPA. Briefly, microsomes were incubated with NADPH and [^3^H] cortisone. The product, [^3^H] cortisol, was specifically captured by monoclonal antibody coupling to protein A-coated SPA beads. IC_50_ (X ± SD, n = 3) values were calculated by using Prism Version 4 (GraphPad Software, San Diego, CA, USA).

## 4. Conclusions

A new ursane-type triterpenoid, 3*β*-hydroxy-urs-30-*p*-*Z*-hydroxycinnamoyloxy-12-en-28-oic-acid (**1**), was isolated from the whole parts of *T. viscidum* together with three known triterpenoids, 3*β*-hydroxy-urs-30-*p*-*E*-hydroxycinnamoyloxy-12-en-28-oic-acid (**2**), 2*α*,3*β*,19*α*-trihydroxy-urs-12-en-28-oic-acid (**3**), ursolic acid (**4**), four known lignans, pinoresinol (**5**), 9*α*-hydroxypinoresinol (**6**), (+)-medioresinol (**7**), (+)-kobusin (**8**), and two steroids, *β*-sitosterol (**9**), daucosterol (**10**). Their structures were established by a combination of spectroscopic data analysis, and comparison with literature data. Compounds **2**–**10** were isolated from *T. viscidum* for the first time. Compounds **1**–**4** were tested for 11*β*-HSD1 inhibition activities *in vitro*, whereby compound **4** showed stronger inhibitory activity, while compounds **1**–**3** did not show obvious 11*β*-HSD1 inhibitory activities.

## Figures and Tables

**Table 1:** ^1^H-NMR (400 MHz) and ^13^C-NMR (100 MHz) Spectral Data of Compound **1** in C_5_D_5_N (*δ* in ppm, *J* in Hz).

NO.	*δ*_H_	*δ*_C_	NO.	*δ*_H_	*δ*_C_
1*α*	1.00 m	39.6	20*β*	1.43 m	44.4
1*β*	1.58 m				
2	1.86 overlap	28.6	21*α*	1.62 m	26.1
			21*β*	1.79 m	
3*α*	3.48 dd (6.8, 9.2)	78.6	22*α*	2.07 overlap	37.4
			22*β*	1.96 overlap	
4		39.9	23	1.26 s	29.3
5*α*	0.88 m	56.3	24	1.05 s	17.0
6*α*	1.59 m	19.3	25	0.92 s	16.2
6*β*	1.39 m				
7*α*	1.57 overlap	34.1	26	1.08 s	17.9
7*β*	1.39 overlap				
8		42.9	27	1.23 s	24.4
9*α*	1.65 m	48.5	28		180.2
10		37.8	29	1.09 d (6.0)	17.6
11*α*	1.64 m	24.1	30a	4.50 dd (3.2, 11.2)	68.2
11*β*	1.97 m		30b	4.27 dd (7.2, 11.2)	
12	5.52 t (3.2)	126.7	1'		167.5
13		139.3	2'	6.09 d (12.4)	116.9
14		40.5	3'	7.03 d (12.4)	144.5
15*α*	1.25 overlap	29.1	4'		127.1
15*β*	2.35 m				
16*α*	2.14 m	25.4	5', 9'	8.09 d (8.4)	134.0
16*β*	2.04 m				
17		48.3	6', 8'	7.22 d (8.4)	116.5
18*β*	2.7 d (11.6)	53.8	7'		161.1
19*α*	1.84 overlap	34.9			

## Supplementary Materials

Supplementary materials can be accessed at: http://www.mdpi.com/1420-3049/18/1/1262/s1.
