# Supplementary material for: A New Triterpenoid from Teucrium viscidum
Source: Molecules. 2013 Jan 21;18(1):1262–9. doi: 10.3390/molecules18011262 (PMC6270140; doi:10.3390/molecules18011262)

Supporting Information

Figure S1. UV spectrum of compound **1** (THF);
Figure S2. IR spectrum of compound **1**;
Figure S3. HRESIMS spectrum of compound **1**;
Figure S4. ^{1}H-NMR spectrum of compound **1** (400MHz Pyridine-d_5);
Figure S5. ^{13}C-NMR spectrum of compound **1** (100MHz Pyridine-d_5);
Figure S6. DEPT spectrum of compound **1**;
Figure S7. HSQC spectrum of compound **1**;
Figure S8. HMBC spectrum of compound **1**;
Figure S9. ^{1}H-^{1}H-COSY spectrum of compound **1**;
Figure S10. NOESY spectrum of compound **1**.

Figure S1. UV spectrum of compound **1** (THF).

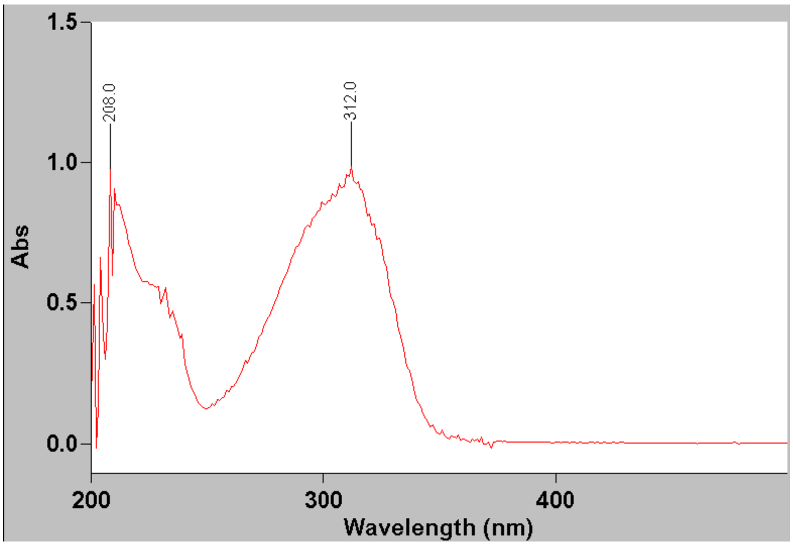

Figure S2. IR spectrum of compound **1**.

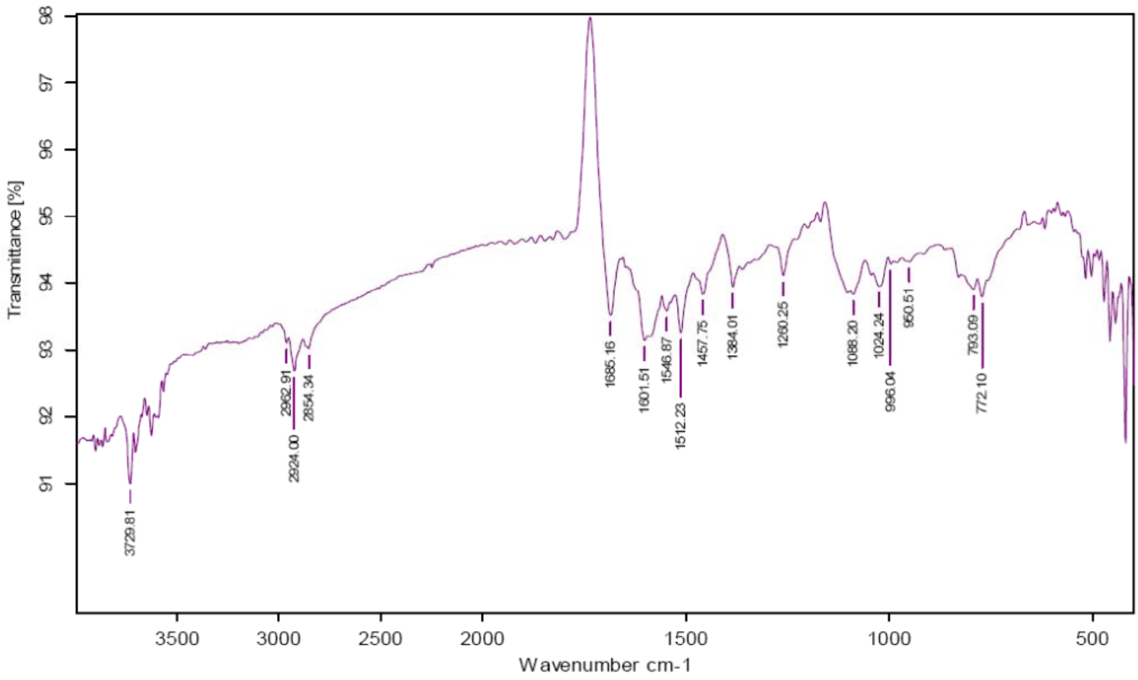

Figure S3. HRESIMS spectrum of compound **1**.

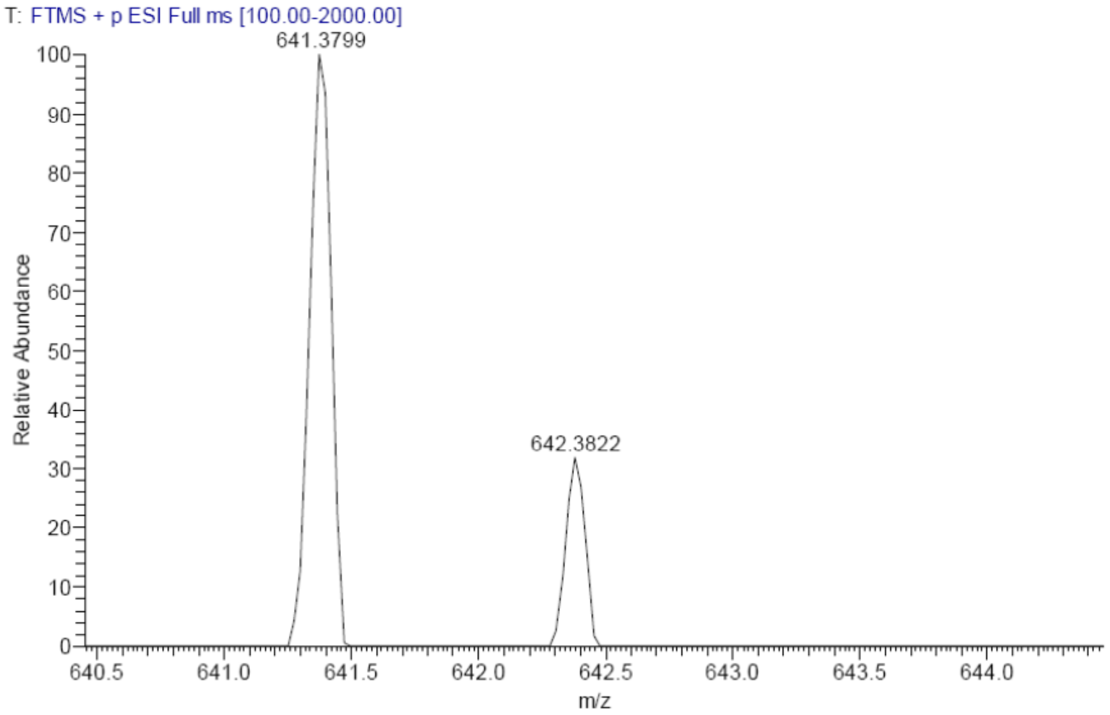

Figure S4. ^{1}H-NMR spectrum of compound **1** (400 MHz Pyridine-d_5).

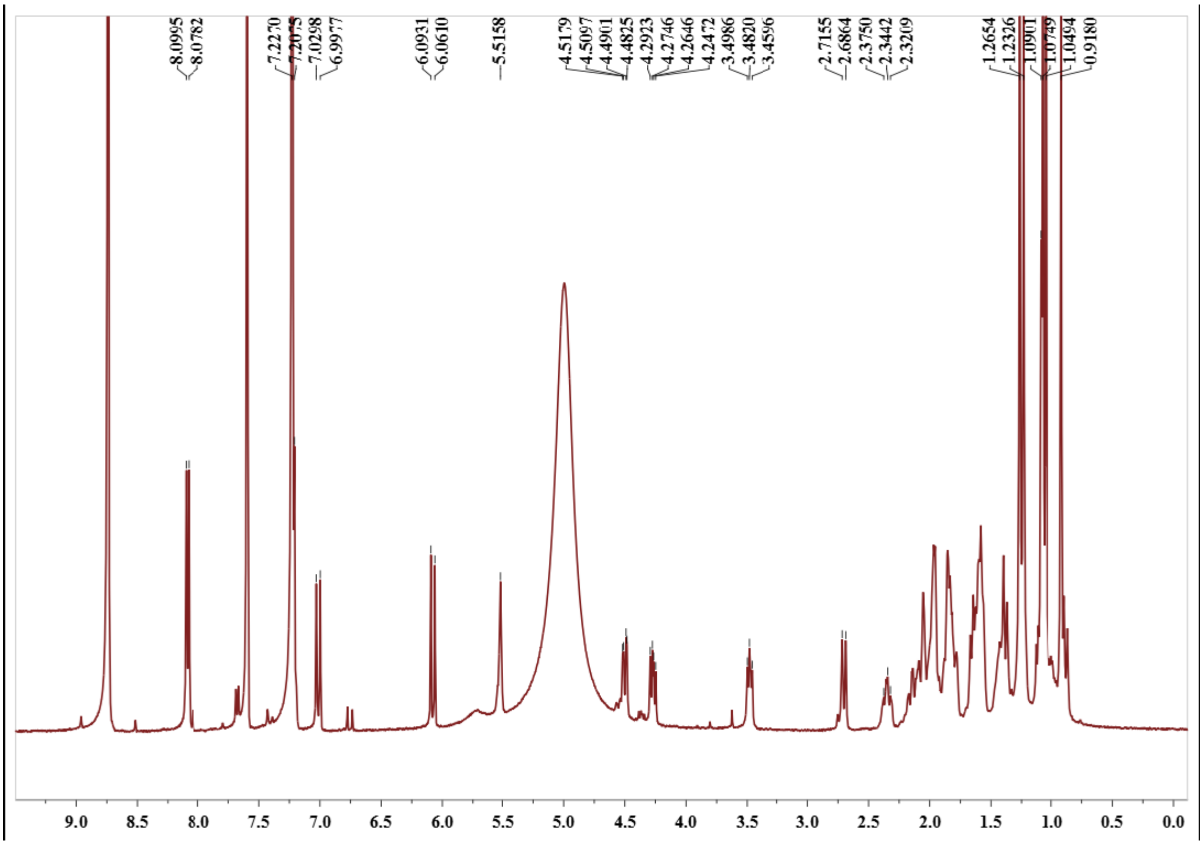

Figure S5. ^{13}C-NMR spectrum of compound **1** (100 MHz Pyridine-d_5).

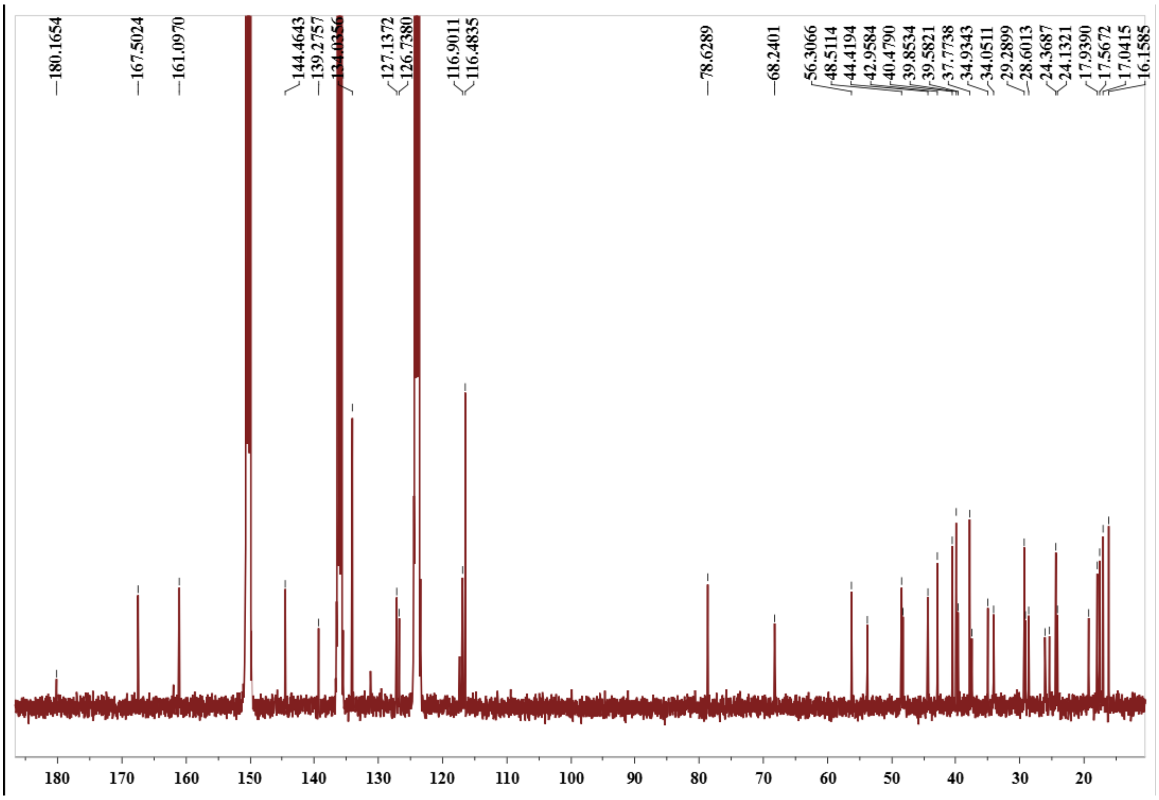

Figure S6. DEPT spectrum of compound **1**.

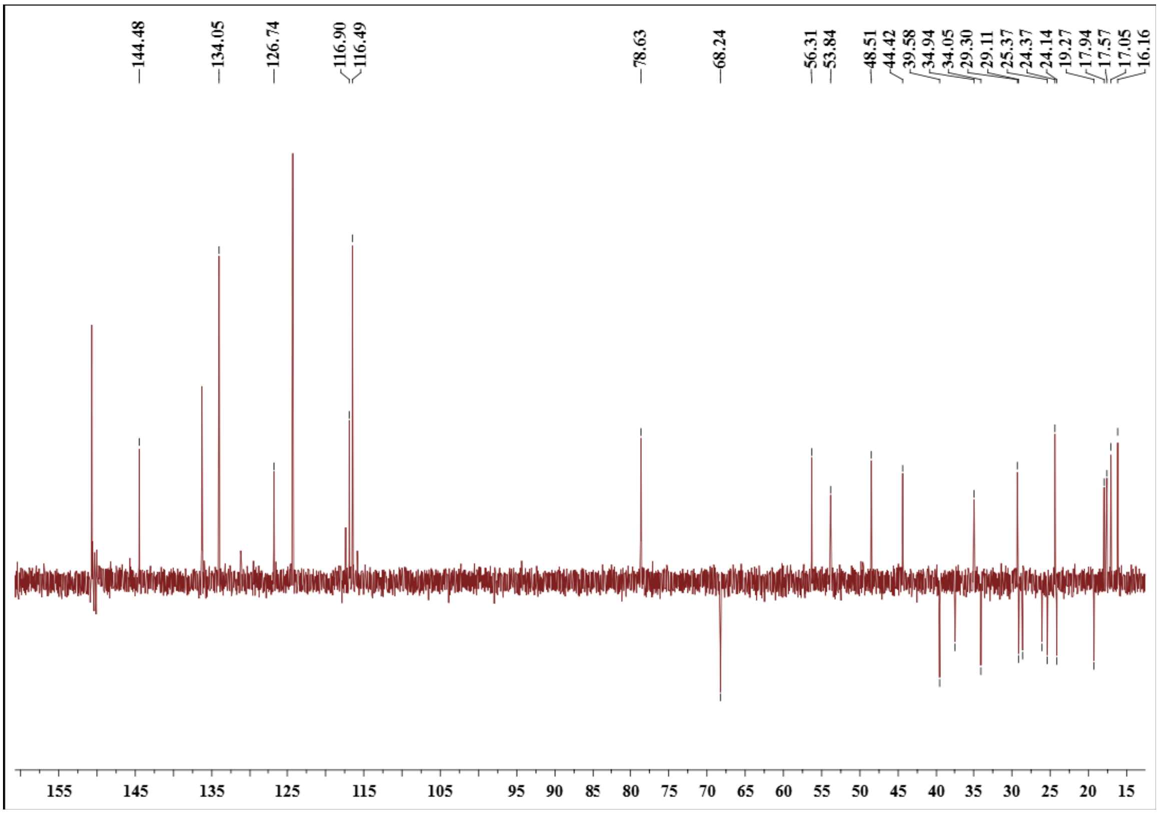

Figure S7. HSQC spectrum of compound **1**.

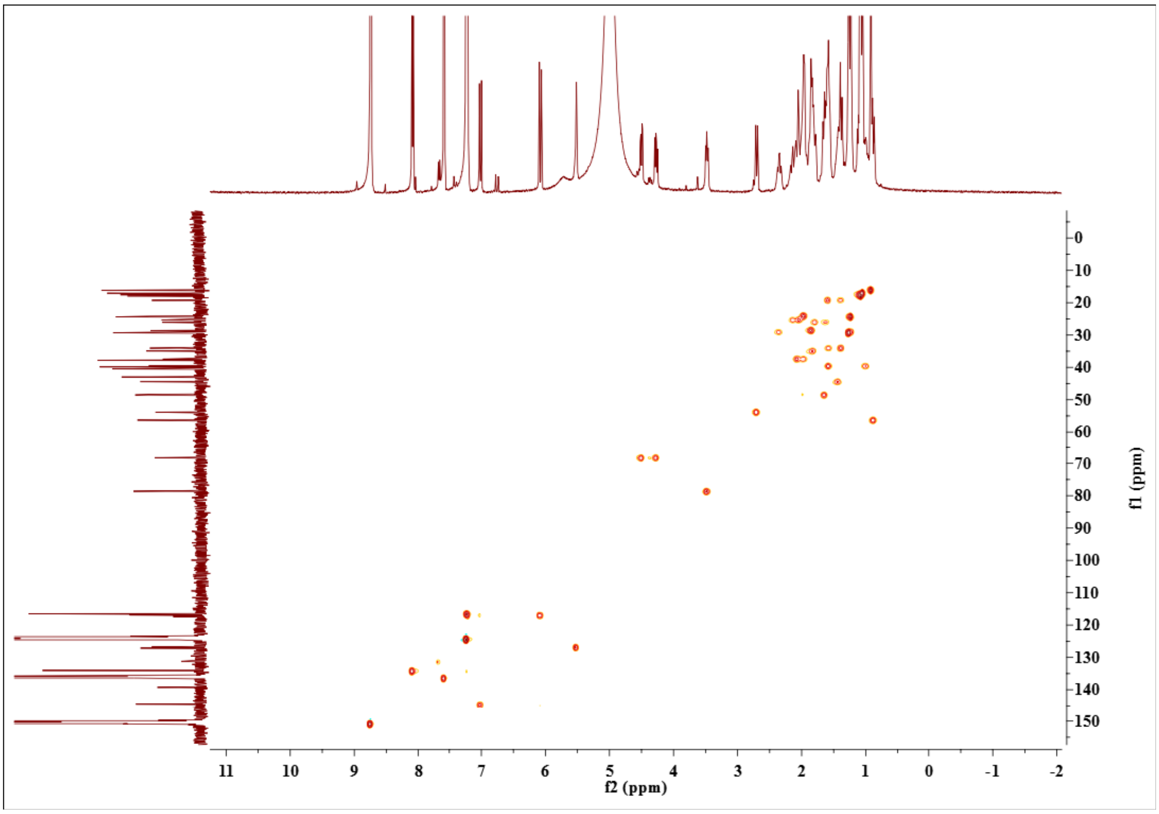

Figure S8. HMBC spectrum of compound **1**.

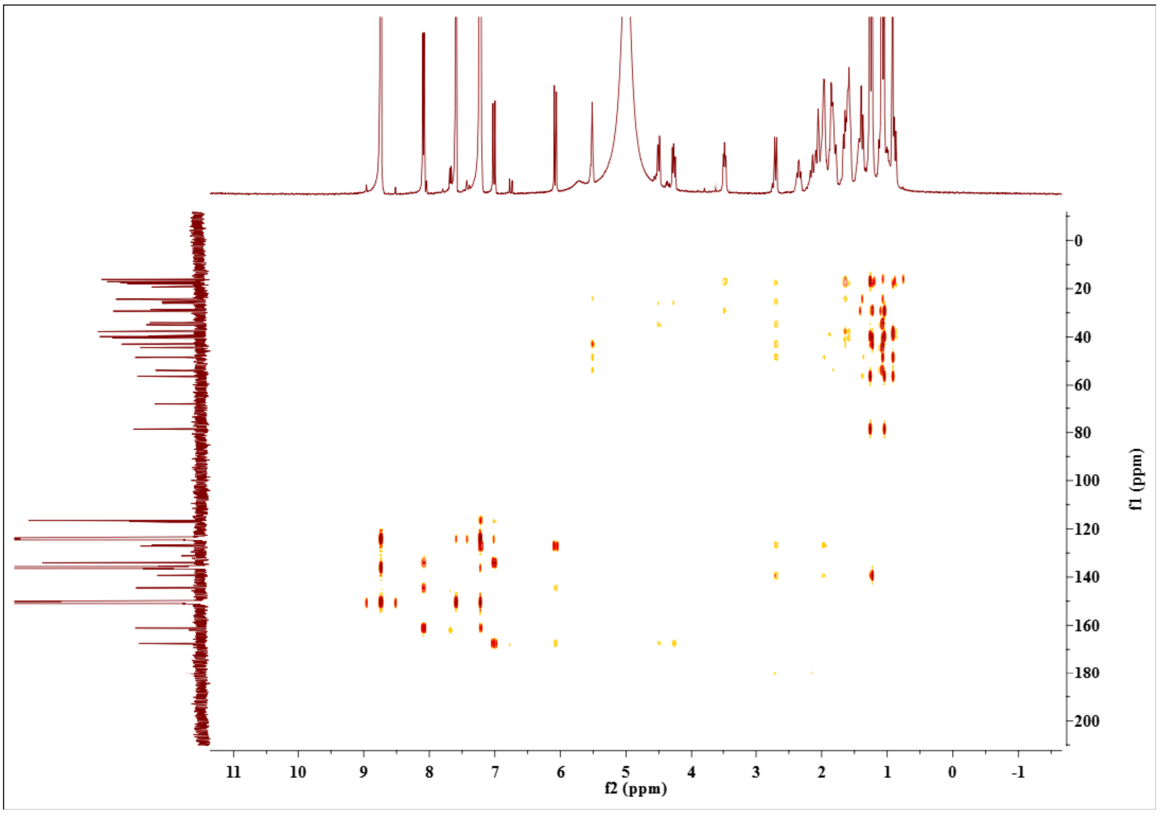

Figure S9. ^{1}H-^{1}H COSY spectrum of compound **1**.

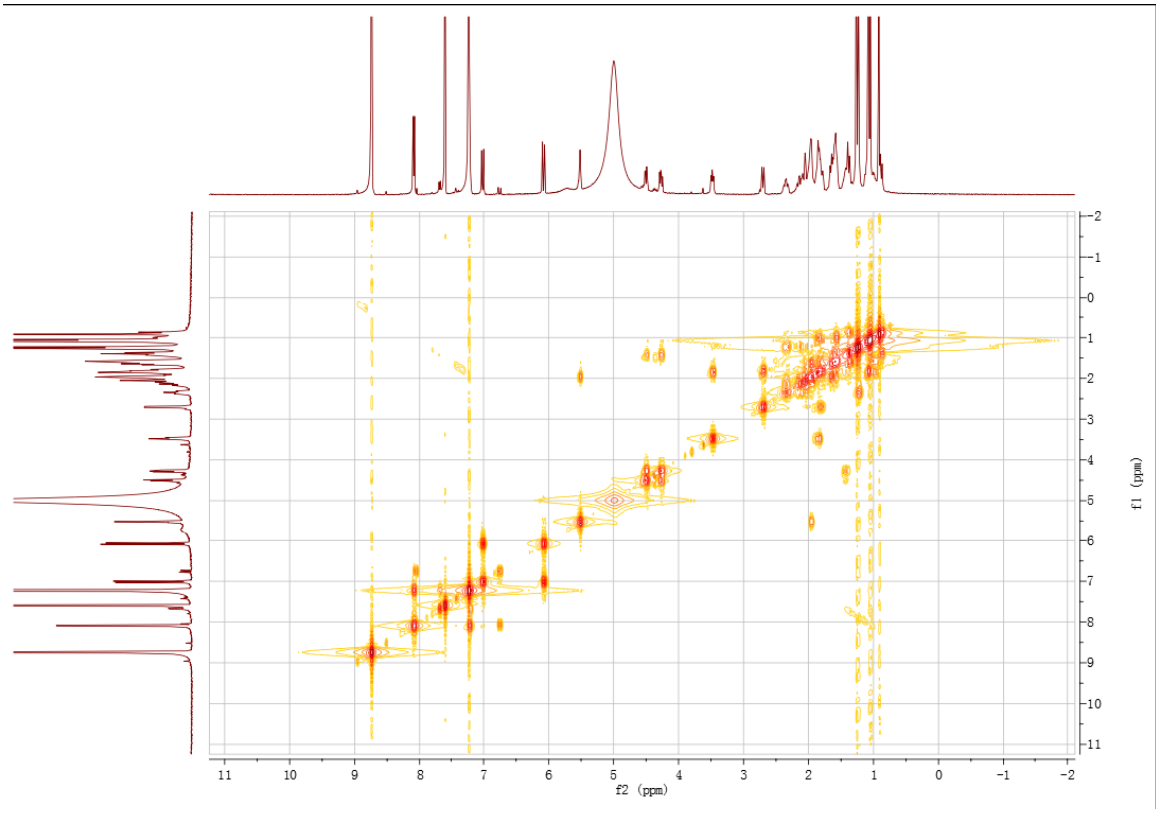

Figure S10. NOESY spectrum of compound **1**.

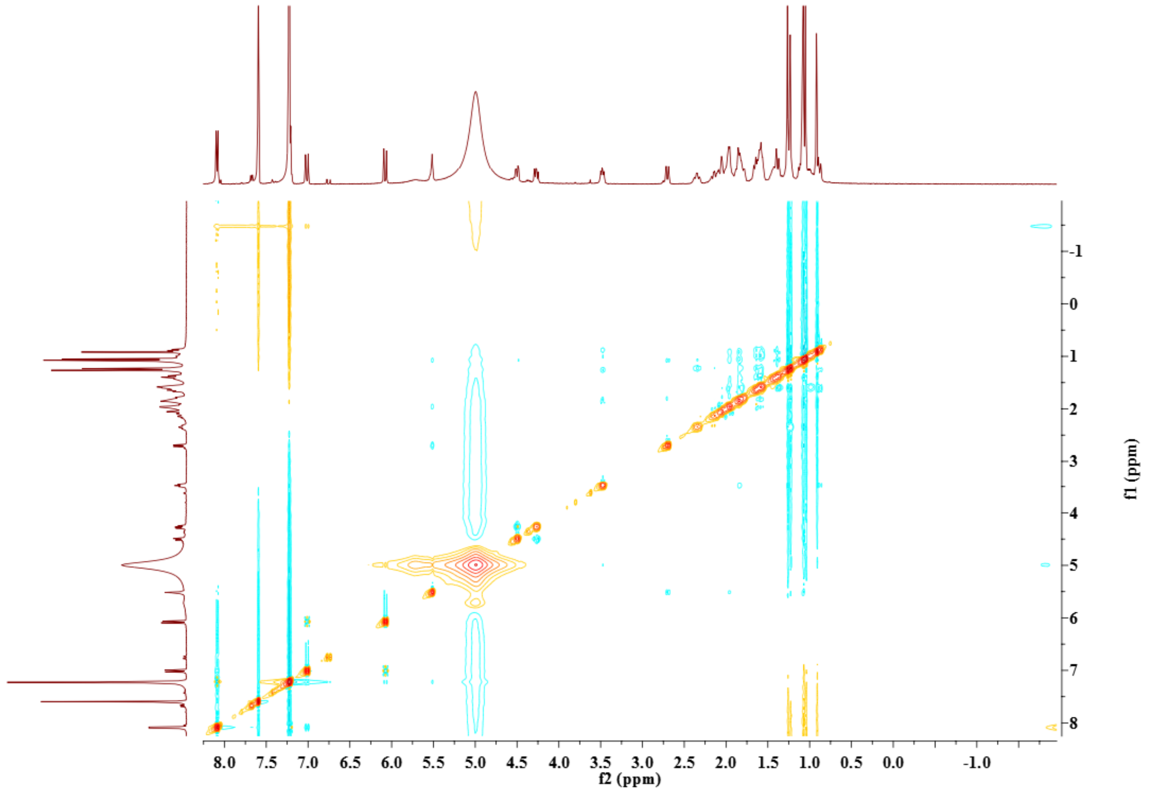

Supplement: Supplementary file 1 [file molecules-18-01262-s001.pdf]
